# The Response of *Thalassiosira pseudonana* to Long-Term Exposure to Increased CO_2_ and Decreased pH

**DOI:** 10.1371/journal.pone.0026695

**Published:** 2011-10-28

**Authors:** Katharine J. Crawfurd, John A. Raven, Glen L. Wheeler, Emily J. Baxter, Ian Joint

**Affiliations:** 1 Plymouth Marine Laboratory, Plymouth, United Kingdom; 2 Division of Plant Sciences, University of Dundee at the James Hutton Institute, Dundee, United Kingdom; Institute of Marine Research, Norway

## Abstract

The effect of ocean acidification conditions has been investigated in cultures of the diatom *Thalassiosira pseudonana* CCMP1335. Expected end-of-the-century pCO_2_ (aq) concentrations of 760 µatm (equivalent to pH 7.8) were compared with present-day condition (380 µatm CO_2_, pH 8.1). Batch culture pH changed rapidly because of CO_2_ (aq) assimilation and pH targets of 7.8 and 8.1 could not be sustained. Long-term (∼100 generation) pH-auxostat, continuous cultures could be maintained at target pH when cell density was kept low (<2×10^5^ cells mL^−1^). After 3 months continuous culture, the C:N ratio was slightly decreased under high CO_2_ conditions and red fluorescence per cell was slightly increased. However, no change was detected in photosynthetic efficiency (F_v_/F_m_) or functional cross section of PS II (σ_PSII_). Elevated pCO_2_ has been predicted to be beneficial to diatoms due to reduced cost of carbon concentration mechanisms. There was reduced transcription of one putative δ-carbonic anhydrase (*CA-4*) after 3 months growth at increased CO_2_ but 3 other δ-CAs and the small subunit of RUBISCO showed no change. There was no evidence of adaptation or clade selection of *T. pseudonana* after ∼100 generations at elevated CO_2_. On the basis of this long-term culture, pH change of this magnitude in the future ocean may have little effect on *T. pseudonana* in the absence of genetic adaption.

## Introduction

Anthropogenic emissions of carbon dioxide (CO_2_) are leading to rapid changes of ocean pH. This is commonly referred to as ocean acidification [1] because, as CO_2_ dissolves in the surface ocean, aqueous carbon dioxide (CO_2_ (aq)) reacts with water to form carbonic acid (H_2_CO_3_), which is a weak acid. This change in the surface ocean is potentially important for phytoplankton not only because the pH of the growing medium (seawater) changes, but also because there will be an increase in the concentration of CO_2_ (aq) – the substrate for RUBISCO. The implications of these changes for marine phytoplankton are not yet clear and a number of studies to date indicate conflicting responses.

The pH of the surface ocean is expected to decrease from an average present-day pH of ∼8.1, to ∼7.8 by the end of the century [1]. Coccolithophores, as calcifying organisms, are one phytoplankton group that has been a major focus for experiments to investigate the consequences of ocean acidification because they may be particularly vulnerable to pH change. A pH of 7.8 is close to the saturation state (Ω) for calcite, leading to increased dissolution. In one of the first studies on the effects of ocean acidification, Riebesell and colleagues [2] showed that growth at 780–850 µatm pCO_2_ resulted in malformed coccoliths of *Emiliana huxleyi* and *Gephyrocapsa oceanica*. However, subsequent studies have shown inconsistent effects of reduced pH and Ridgwell and colleagues [3] concluded that it was difficult to draw consistent conclusions about the effect of pH change on coccolithophores and suggested that inter- and intra-species differences may be responsible for the range of observed effects. More recently, it has been suggested that different nutrient and light regimes may account for the observed variability in these experimental studies [4]. A global study of individual coccolith mass in ocean sediments from the past forty thousand years broadly supports the hypothesis that calcification is likely to decline with a decrease in ocean CO_3_^2−^ concentrations [5].

The effect of higher pCO_2_ on other phytoplankton groups is also unclear. From a theoretical point of view, Raven [6] suggested that increased CO_2_ (aq) availability should benefit those phytoplankton species that rely on diffusive CO_2_ entry, or those that are able to suppress carbon concentrating mechanisms (CCMs). Those species that depend on diffusive entry of CO_2_ may have higher intracellular CO_2_ (aq) concentrations that are closer to those required to saturate RUBISCO. Higher CO_2_ (aq) should reduce photorespiration in species with low specificity RUBISCO, since high CO_2_:O_2_ concentrations should result in an increased carboxylase:oxygenase ratio [7]. This may be particularly beneficial under low light, growth-limiting conditions. However, most phytoplankton use CCMs and actively take-up both CO_2_ (aq) and HCO_3_^−^ [8], [9]. Therefore, the species that may benefit from a reduction in CCM activity could come from any phytoplankton group. A reduction of CCM mechanisms, i.e. fewer CCM proteins, would reduce the nitrogen requirement for CCM protein synthesis and could result in a concomitant reduction in the energy requirements for protein synthesis [6].

If there are benefits in terms of energetic costs to the cell, are these of sufficient magnitude to influence growth rate? That is, would higher CO_2_ (aq) appear to act as a fertilizer? Again, current information is contradictory, although we perhaps should not assume a uniform response in all diatom species. For example, metabolic diversity in diatoms, such as the proposed C4 mechanism for C fixation in certain species [9], may lead to diverse responses to elevated CO_2_. Laboratory cultures of *Thalassiosira pseudonana* exposed to a range of CO_2_ concentrations showed no increase in specific growth rate when dissolved CO_2_ exceeded the atmospheric equilibrium concentration of 15 µM CO_2_ [10]. Saturation at the air-equilibrium CO_2_ is consistent with the data of Clark and Flynn [11] and of Roberts et al. [12] on *T. pseudonana* and *Thalassiosira weissflogii*. In an experimental mesocosm bloom, a 40% increase in the specific growth rate of *Skeletonema costatum* was observed at 750 µatm compared to 250 µatm and 400 µatm pCO_2_ [13]; in contrast, there was no effect on growth rate of *Nitzschia* spp. Also, Beardall and Raven [7] found a 24% increase in specific growth rate of the diatom *Chaetoceros muelleri* at 1000 µatm pCO_2_. In experiments with natural assemblages, increased growth rates were observed at 800 µatm pCO_2_ compared with 380 µatm pCO_2_ in three separate incubations of summer assemblage diatoms from the Ross Sea [14]. However, this does not appear to be a consistent response because the same treatment of a *Thalassiosira* spp.-dominated-community in the Californian upwelling did not alter specific growth rate [15].

In this study, we investigated the effect of higher CO_2_ (aq) on the diatom, *Thalassiosira pseudonana.* Diatoms are very important for the productivity of the oceans, contributing ∼45% of global marine primary production [16]. In addition, the genome of *T. pseudonana* has been fully sequenced and annotated [17]. A major difficulty with laboratory culturing approaches is to maintain constant pH and CO_2_ (aq). As phytoplankton photosynthesize and culture cell density increases, CO_2_ (aq) is removed from the medium, leading to a rapid increase in pH; a change of 1 pH unit in a few days is not unusual. Organic buffers would appear to be an obvious approach to control variations in pH. However, organic buffers can have adverse effects on physiology and, in combination with aeration at different concentrations of CO_2_, can have a profound effect on the total DIC, acting to increase carbonate at lower seawater pH [18]. Organic buffers may also form complexes with metals, reducing availability to phytoplankton [19]. Organic buffers are not a panacea.

We have used low cell density, continuous cultures to overcome these problems, and to maintain constant growth conditions for a long-term investigation. We have employed a pH auxostat in which pH is constantly monitored and maintained by the influx of fresh media. Auxostats differ from chemostats in that dilution rate is not constant and is self-regulated by metabolic activity within the culture. Cell density in a pH auxostat is therefore determined by the buffering capacity of the input media and the stoichiometry of H^+^ production/consumption in relation to growth rate and can be defined by the following equation:
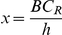



where x is cell density, BC_R_ is the buffering capacity of the input media and h is the H^+^ consumption or generation relative to growth rate [20]. The pH auxostat approach has many advantages over batch culture for maintaining carbonate chemistry for long periods of time.

This approach was capable of maintaining stable pH for a period of 3 months (∼100 generations). The aim was to use long-term cultures, growing under constant conditions, to test if acclimation or adaptation to higher CO_2_ (aq) might occur. By acclimation, we mean that the organism has sufficient metabolic flexibility to be able to grow under higher CO_2_ (aq) conditions; adaptation would imply selection of mutant clades that could survive in higher CO_2_ (aq) but not at air-equilibrium CO_2_ (aq), or even that have increased fitness during growth at higher CO_2_ (aq) ([21]–[23]; also see Huertas and colleagues [24] for results on the related effect of warming). In order for adaptation to present within a population of cells, a mutation which confers a fitness advantage has to become present at a sufficient frequency within a population in order to convey a phenotypic change. In reality this may take many hundreds of generations and many more so for mutations which convey only a very subtle increase in fitness [25]. For ocean acidification experiments using eukaryotes (with generation times ranging from 0.5 to several days) and where carbonate chemistry must be precisely controlled throughout the duration of the study, this clearly represents a formidable challenge. The required number of generations to observe adaptation is dependent on many variables including the number of cells in each population and the fitness advantage conferred by a specific mutation. It is unlikely that 100 generations will be sufficient to examine for adaptation within conventional batch culturing approaches, although in continuous cultures the rapid wash out of slow growing strains may allow adaptation to present more rapidly within a population. Nevertheless, it remains a valid hypothesis to test.

We have investigated a number of cellular characteristics, such as carbon and pigment content, and focused specifically on expression of carbonic anhydrases as components of CCMs and also of diffusive entry of CO_2_. Carbonic anhydrases catalyze the equilibrium of inorganic carbon species – the reversible hydration of CO_2_ to HCO_3_^−^ - and are assumed to ensure a high rate of supply of CO_2_ to the low affinity active site of RUBISCO within the chloroplast. We selected 4 δ-carbonic anhydrase (δ-CA) from the *T. pseudonana* genome, since McGinn and Morel [26] have demonstrated significant changes in δ-CA transcripts in response to changes in carbon availability. In addition, down-regulation of these enzymes in response to higher CO_2_ (aq) might result in savings in energy and N-resources that might be detectable as a change in cellular physiology. Batch cultures experiments were used to distinguish between adaptation and acclimation after growth for >100 generations at the higher, and the present CO_2_ (aq) concentrations. Inocula from both long-term CO_2_ (aq) conditions were used to initiate batch cultures in both CO_2_ treatments and cell properties were determining. Acclimation was assumed if the properties of cells grown for >100 generations in higher CO_2_ (aq) reverting to the properties found for the lower CO_2_ (aq) >100 generation culture, while adaptation would be demonstrated by retention of characteristics shown after 100 generations of growth at high CO_2_ (aq) when the cells are subsequently cultured at the lower CO_2_ (aq) (see [23]).

## Materials and Methods

### Continuous cultures

An axenic culture of the diatom *Thalassiosira pseudonana* CCMP1335 (Coscinodiscophyceae) (Hustedt) Hasle et Heimdal was obtained from the Provasoli-Guillard National Center for Culture of Marine Phytoplankton (CCMP), Maine, USA. Cultures were grown in f/2 + Si media [27], based on seawater collected from a long-term observatory in the English Channel ([28]; www.westernchannelobservatory.org.uk). Axenic cultures were grown at 14°C with a light:dark cycle of 16∶8 h. Light was cool-white fluorescent, resulting in a photon flux of 60 µmol quanta m^−2^ s^−1^ in the centre of each culture vessel.

There were two treatments – higher CO_2_ (aq) (760 µatm CO_2_) and present-day CO_2_ (aq) (380 µatm CO_2_) conditions. Media and cultures for present-day conditions were bubbled with 0.2 µm filtered air, resulting in a pH of 8.1. Media for the higher CO_2_ (aq) cultures was equilibrated with a gas mixture of air and CO_2_, with a certified concentration of 760 µatm CO_2_ (BOC, Special products, Guildford, UK), resulting in a pH of 7.8. Media were equilibrated with the appropriate 0.2 µm-filtered gas phase immediately after autoclaving for a minimum of three days and equilibration was determined by measuring pH at regular intervals until it remained constant. Three replicate cultures for each treatment were gently aerated with sintered glass diffusers at the bottom of the vessels at a rate of approximately 40 mL min^−1^. For continuous cultures, medium was pumped into the culture in response to a change in culture pH detected by an Aquatronica, (Aquatronica, Cavriago, Italy) aquarium controller (**Figure S1**). When the pH deviated by 0.01 pH units from the set value, fresh pre-equilibrated medium was pumped into the culture vessels. Cultures were stirred continuously with magnetic stir bars at low speed. Culture volume was maintained at 350 mL, exit gas carrying media up a waste tube when the culture volume exceeded 350 mL. Exit gas was vented through a port fitted with a 0.2 µm filter.

### Determination of cell number and characteristics by flow cytometry

Cells were counted and light scatter and autofluorescence was determined using a Becton Dickinson FACScan benchtop flow cytometer (Becton Dickinson, Oxford, UK), fitted with 15 mW air-cooled lasers exciting at 488 nm and standard filter combinations. Light scattered relates to a particles' refractive index, size and shape; auto-fluorescence is a measure of chlorophyll content [29]. CELLQuest v3.0 software (Becton Dickinson) was used to analyse the data. The flow rate was calibrated using fluorescent microspheres of a known concentration (Beckman Coulter flow set fluorospheres), and means of triplicate analyses were calculated. The specific growth rate per day (µ) was calculated as 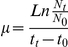



where *N* is the number of cells at time t_1_ and t_0_ (in days).

pH was measured using a Mettler Toledo MP220 pH meter Calibrated with pH 4, 7 and 10 standards. It has been suggested [30] that the high nutrient concentration of culture media complicates the determination of carbon dioxide speciation. Therefore we relied on the certified air/CO_2_ mixtures, rather than determination of pH, total alkalinity or dissolved inorganic carbon determinations to control the experimental conditions.

### Physiological assessment

Fv/Fm and σ_PSII,_ were measured using a SATLANTIC Fluorescence Induction and Relaxation (FIRe) (Satlantic, Halifax, CA). Samples were held in the dark for 30 min, 3 mL samples were loaded in the dark and diluted with fresh f/2 media as necessary. Fluorescent blanks were estimated using 0.2 µm filtered media.

Carbon and nitrogen concentrations were determined using a Thermoquest Flash EA1112, elemental analyser. Samples were filtered through 25 mm GF/C glass fibre filters (Whatman), prepared by ashing for 4 h at 450°C to remove organic contamination. Sample volume was adjusted to yield approximately 7 µg of nitrogen per sample. Filters were dried and stored until analysed.

### qPCR analysis of gene expression

The cells were harvested by centrifugation 2 h after the onset of the light period. The cell pellets were ground in a pestle and mortar with liquid nitrogen and RNA was extracted using an RNeasy mini kit with on-column DNAse treatment (both Qiagen). RNA quality was assessed using an Agilent 2100 Bioanalyser and the first strand cDNA was reverse transcribed using oligo d(T)_16_ primers. The primers used for qPCR are listed in **Table S1**. The terminology to identify different CA genes follows that used by Tachibana and colleagues [31]. Each qPCR reaction mix contained 10 ng of cDNA, optimised concentrations of the appropriate primers and 25 µL of Power SYBR® Green PCR Master Mix in a total reaction volume of 50 µL. Amplification was performed using an ABI PRISM 7000 (Applied Biosystems, Foster city, USA) with the following thermal cycling conditions: 10 min at 95°C, followed by 40 cycles of 15 s at 95°C and 1 min at 60°C. Duplicate reactions were run for each RNA extraction. Dissociation analysis was performed following the amplification to confirm that a single product was amplified. Data were analysed using Relative Expression Software Tool (REST) 2008 software (www.gene-quantification.info) which enables the use of multiple housekeeping genes for normalisation and incorporates a pair-wise fixed randomisation test [32]. Two housekeeping genes, *EF-1α* and β*-actin* demonstrated a consistent level of expression relative to each other in both pH auxostat and batch cultures harvested at different pH and cell densities. The expression of the target genes (δ-carbonic anhydrases *CA-4* to *CA-7* and RUBISCO small subunit, *rbcS*) from pH auxostat cultures at 760 µatm CO_2_ was determined relative to those at 380 µatm CO_2_. Unfortunately, the RNA samples harvested from pH auxostat 5 (380 µatm CO_2_) were lost during processing and we were therefore only able to obtain gene expression data from two of the present-day (380 µatm CO_2_) pH auxostats.

### Acclimation/adaptation assays

Following growth in continuous culture for 3 months, the effect of treatment was assessed in batch cultures under the same conditions of light and temperature as the continuous cultures. Triplicate flasks containing 900 mL of 760 µatm and 380 µatm CO_2_ equilibrated media were inoculated from each of the long-term continuous cultures to an initial cell density of 1×10^5^ mL^−1^. Cell numbers were determined daily by Coulter Multisizer II Coulter Counter and pH was also measured. The experimental design involved inoculating media equilibrated at both 760 µatm and 380 µatm CO_2_ with cells from both treatments of the long-term cultures, i.e. 100 generation cells acclimated to both higher CO_2_ (aq) and present-day. That is, cells were transferred from continuous culture both to the same conditions (i.e. 760→760 and 380→380 µatm CO_2_ (aq)), and to the opposite treatment (e.g. 760→380 and 380→760 µatm CO_2_ (aq)). Cells were harvested in mid-exponential phase growth and analysed for C:N content, cell size and pigment content (using flow cytometry). As with all batch cultures, and despite aeration of cultures, pH of the batch cultures increased rapidly as the cell density increased. However, a consistent difference in pH was maintained between the two sets of experimental conditions; a permutation-based analysis of variance (PERMANOVA, a routine of the PRIMER statistics package) confirmed a significant difference in pH was maintained between the 760 µatm and 380 µatm CO_2_ treatments despite changing pH (Pseudo-F = 5.99, p = 0.002).

## Results

### pH control in aerated batch cultures

**Figure 1:**
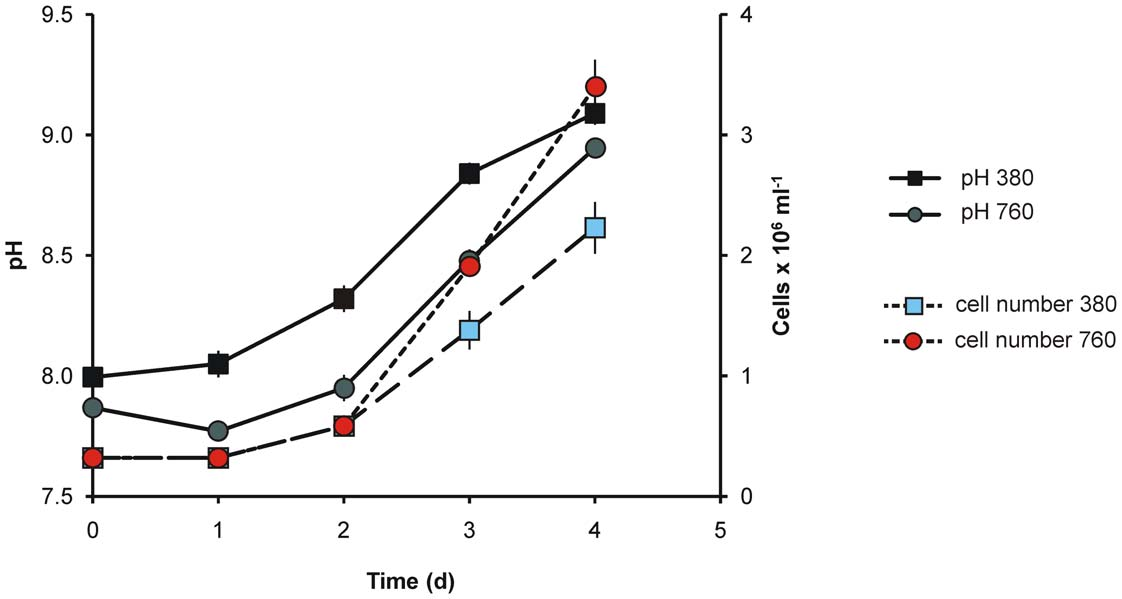
Change in pH of a batch culture of *T. pseudonana* grown in f/2 medium at 15°C. Solid lines are the mean pH values from triplicate cultures aerated throughout the experiments with air at 380 µatm pCO_2_ or air enriched with CO_2_ at 760 µatm pCO_2_. Dashed lines are mean cell number from the same experiment. Error bars are ±2SD.

The ability of *T. pseudonana* cultures to change the carbonate system within the culture media was monitored by measuring pH in batch cultures (f/2 medium). In low cell density cultures (<1.8×10^5^ cells mL^−1^), the pH of cultures bubbled with 760 µatm CO_2_ remained at around pH 7.8 (**Figure 1**). However, at higher cell densities, pH was not controlled and increased rapidly to >9. We concluded, in accordance with similar studies of other phytoplankton, that for *T. pseudonana*, bubbling with CO_2_ can only control pH at very low cell densities.

Organic buffers, such as Tris or HEPES, have also been used to control pH in phytoplankton cultures and reduce variability in culture conditions during long-term experiments. Batch cultures were tested for pH control with Tris concentrations of 0, 4, 8, 16.5 and 33 mmol L^−1^. In all cases, culture pH increased at cell densities >0.7×10^6^ and constant pH was only maintained at initial pH values with the addition of 40 mmol L^−1^ Tris. However, such high buffer concentrations had adverse effects on growth rate of *T. pseudonana* and cell densities of buffered cultures were less than half those of unbuffered controls after 5 days. Specific growth rates (µ) were also reduced. For example, in f/2 media buffered with 40 mmol L^−1^ Tris and aerated with air, cultures had maximum specific growth rates (µ_max_) of 0.93±0.04 d^−1^ and in equivalent unbuffered f/2 medium, µ_max_ was 1.34±0.11 d^−1^. From these findings, we concluded that the addition of organic buffers was not an appropriate methodology to study the long-term effects of ocean acidification on *T. pseudonana*.

Alternative approaches to achieve long-term control of culture pH and carbonate chemistry within *T. pseudonana* cultures are continuous or semicontinuous culturing approaches. We evaluated the latter using semicontinuous cultures that were diluted each day with fresh medium equilibrated to the required CO_2_ concentration. The aim was to maintain low cell numbers (target 2.5×10^5^ cells mL^−1^) to minimise pH changes that were a consequence of high biomass in the cultures; it was also an aspiration to achieve high growth rates (µ = 1.0 d^−1^). Over a period of 20 days, there was no significant difference between the growth rate of 3 replicate cultures grown at 760 µatm CO_2_ with a mean specific growth rate 0.97 d^−1^ (±2SE 0.11) and 3 cultures bubbled with air (µ = 0.92±0.12). As far as could be ascertained from this short-term experiment, no significant variation in µ as a consequence of pCO_2_ (aq) was detectable (F = 0.05, p = 0.81). It was clear that much longer growth periods (>100 generations) would be required to answer questions relating to adaptation or acclimation.

### Long-term continuous cultures

We developed a continuous culture approach to maintain *T. pseudonana* at elevated CO_2_ for several months. The approach used was a pH auxostat, where pH was constantly monitored and maintained by addition of fresh media equilibrated at the required CO_2_ concentration (**Figure S1**). In this system, when cell densities approached those at which CO_2_ utilisation by the phytoplankton exceeded the additional buffering provided by bubbling with air, the addition of fresh media restored target pH, washed out cells and reduced cell density. This approach had the advantage of maintaining a constant cell density and the constant influx of fresh media ensured perturbations in carbonate chemistry were minimised.

**Figure 2:**
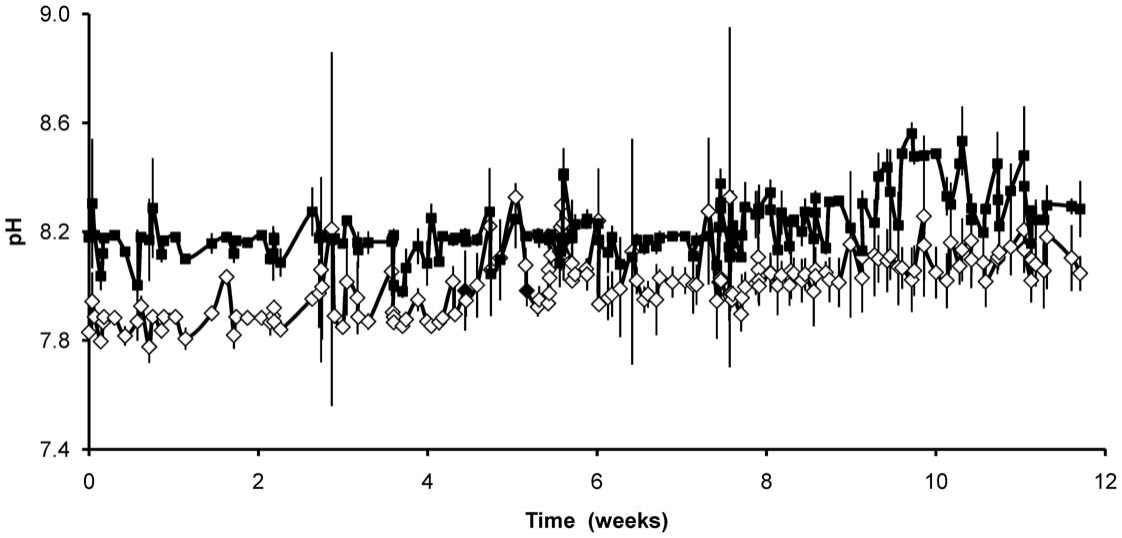
Mean pH of triplicate cultures of *T. pseudonana* grown in pH auxostat continuous culture. Cultures were aerated with either 760 µatm pCO_2_ (◊) or 380 µatm pCO_2_ (▪). Error bars indicate ±2SE.

The dilution method of pH auxostat continuous culture was successful in maintaining constant values of pH over a 3-month period. Variation in pH between triplicate cultures was generally small but, on occasions, there were short-term variations in individual cultures that required operator intervention to correct, due to for example, a misreading pH sensor. These events can be identified (**Figure 2**) as transient increases in the standard errors of the mean. In general, the triplicate cultures of the two treatments responded in the same way over the duration of the experiment. Interestingly, the mean cell density of *T. pseudonana* cultures grown at elevated CO_2_ (760 µatm) was significantly lower than those grown in air (380 µatm CO_2_) (1.91×10^5^ cells mL^−1^ compared to 2.98×10^5^cells mL^−1^ at the end of the 3-month period of growth). At elevated CO_2_, the lower seawater pH and lower concentration of carbonate (CO_3_^2−^) and borate (B(OH)_4_^−^) ions, reducing the acid-base buffering capacity, as well as reducing the buffering of increases in CO_2_ (Revelle Factor) of the seawater [33], [34]. Reduced buffering capacity resulting in increased dilution is the likely cause of the lower cell densities in the pH auxostat continuous cultures at elevated CO_2_, although changes in the H^+^ consumption rate (which is largely due to photosynthetic C uptake) may also contribute.

### Effects of long-term culture on cell physiology

After 3 months of continuous culture, a number of physiological variables were measured (**Table 1**) to assess if there had been any significant change as a consequence of growth at different CO_2_ (aq) and pH. No differences were detected in the general physical properties of the cells, such as the cellular dimensions and cell-surface properties as assessed by flow cytometry. Mean values for forward and side scatter were identical for the triplicate cultures grown under the two treatments after 100 generations. Side scatter was 41±1 (arbitrary units related to laser detection) for the cultures grown at 380 µatm pCO_2_, and 41±3 for the cells grown at 760 µatm pCO_2_. Values of forward scatter were 42±1 for cells grown at 380 µatm pCO_2_ and 42±2 at 760 µatm pCO_2_. Although no change in morphological properties of the cells was detected over the time course of the experiment, the carbon and nitrogen ratio did indicate differences between the two treatments. The mean C:N ratio for cells grown at 760 µatm pCO_2_ was 6.94±0.21, but for cultures grown at 380 µatm pCO_2_, the C:N ratio was 7.47±0.47 (F = 9.18, p < 0.01). That is, higher pCO_2_ (aq) appeared to have resulted in lower C:N ratio in cultures maintained under higher pCO_2_ (aq). However, it should be noted that one of the long-term present-day cultures was more variable than the others and this culture was responsible for the higher SE of the C:N ratio.

**Table 1:** Mean and standard deviations from three continuous cultures for each treatment (380 and 760 µatm pCO_2_) of flow cytometer readings for red fluorescence (Red fl), side scatter (SSC) and forward scatter (FSC) (arbitrary units per cell), Fv/Fm, σ_PSII_, C:N.

	Red fl cell^−1^	SSC cell^−1^	FSC cell^−1^	Fv/Fm	σ_PSII_	C:N
**760 µatm pCO_2_**	251±23	41±3	42±2	0.60±0.02	772±65	6.94±0.21
**380 µatm pCO_2_**	235±4	41±1	42±1	0.62±0.01	742±52	7.46±0.47

Chlorophyll content of each culture, as assessed from red autofluorescence cell^−1^ was significantly higher and more variable (250±23 arbitrary units) for cells grown at 760 µatm pCO_2_, compared to (235±4) for cultures grown under present-day conditions of 380 µatm pCO_2_ (F = 5.23, p = 0.04). Cell density in both treatments and all replicates was low and self-shading was minimal and consistent, so variation in chlorophyll concentration was not due to variation in photon flux density in the 2 cultures treatments. Again, as for C:N ratio, there was some variability in apparently identical continuous cultures and one of the higher CO_2_ (aq) triplicate cultures did not show any increase in red fluorescence cell^−1^. An increase in the chlorophyll cell^−1^ might be expected to change the photosynthetic capacity of the cells. However, no significant differences were detected in measurements of photophysiology. Photosynthetic efficiency (F_v_/F_m_) was 0.62±0.01 for cultures grown at 380 at µatm pCO_2_, and 0.60±0.02 for 760 µatm pCO_2_. No change was observed in the functional absorption cross section of photosystem II (σ_PSII_) (742±52 for 380 µatm pCO_2_ cultures and 772±65 at 760 µatm pCO_2_).

### CCM transcription

**Figure 3:**
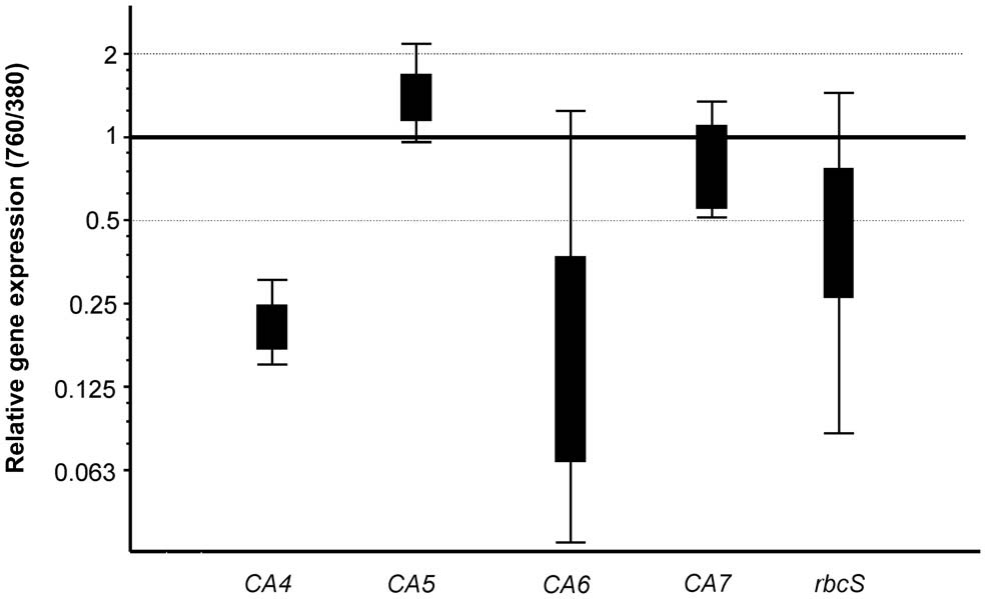
Gene expression of δ-CA and RUBISCO in *T. pseudonana* grown in pH auxostat continuous culture. qPCR was used to determine the gene expression ratio in cultures grown at 760 µatm pCO_2_ relative to those grown at 380 µatm pCO_2_. Boxes represent the inter-quartile range, or the middle 50% of observations. The dotted line represents the median gene expression. Whiskers represent the minimum and maximum observations.

The effect of long-term growth at elevated CO_2_ on components of the CCM was examined by determining the relative expression of four δ-CA homologues (*CA-4* to *CA-7*) and the RUBISCO small subunit, *rbcS* using qPCR, with normalization to two housekeeping genes (*EF-1α* and *β-actin*). Expression of *CA-4* was significantly lower in cultures grown at 760 µatm CO_2_ relative to cultures grown at 380 µatm pCO_2_ (**Figure 3**), whilst *CA-5, CA-7* and *rbcS* did not show any significant differences in gene expression. Expression of *CA-6* also appeared to be slightly lower in 760 µatm pCO_2_ cultures, but this was not statistically significant.

### Acclimation/adaptation assays

**Figure 4:**
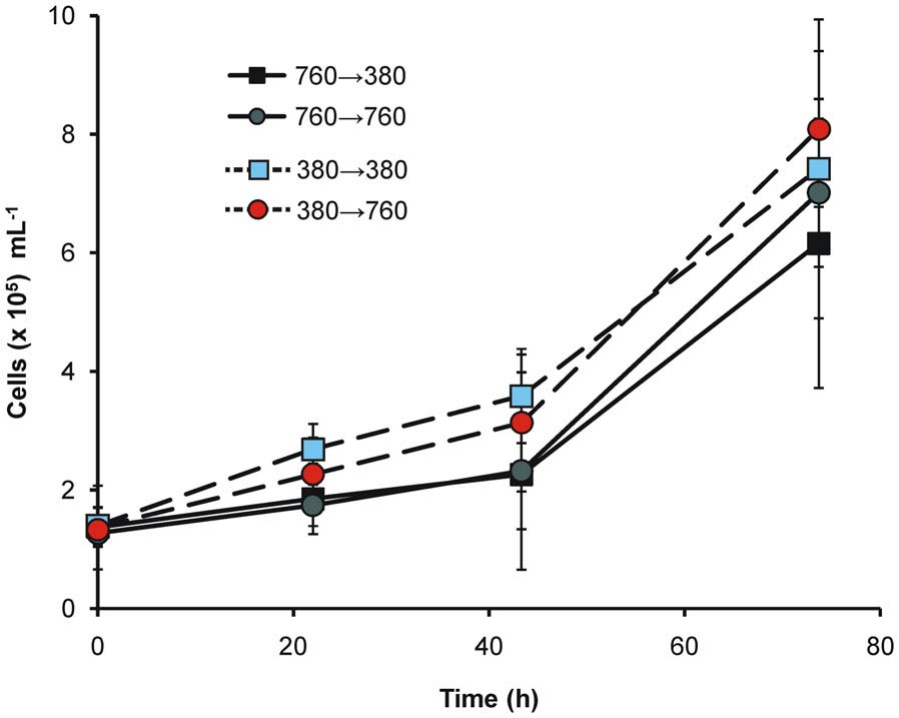
Response in batch culture of *T. pseudonana* cells from long-term, pH auxostat continuous cultures on transfer to different pCO_2_. Cells from all 6 long-term cultures each inoculated into batch cultures aerated at 760 µatm pCO_2_ or 380 µatm pCO_2_. For example, 760→760 represents cells from the long-term culture grown at 760 µatm pCO_2_ inoculated into a batch culture equilibrated with 760 µatm CO_2_. 760→380 represents the same cells (from 760 µatm CO_2_ continuous culture) inoculated into a batch culture equilibrated with 380 µatm CO_2_. Mean values (±2SE) of triplicate batch cultures from each inoculum.

To test for evidence of culture acclimation or adaptation after growth at 760 µatm pCO_2_ for ∼100 generations, cells from the long-term continuous cultures were used to inoculate batch cultures aerated with either 380 µatm or 760 µatm pCO_2_. Batch cultures were used in this assay as growth rates can be easily calculated to provide a simple and robust measure of fitness. Following short lag phases, the cultures grew exponentially (**Figure 4**). Mean specific growth rates (d^−1^) were 0.88±0.29 in the 380→380 treatment, 0.64±0.13 in the 380→760 treatment, 0.91±0.33 in the 760→380 treatment and 1.02±0.19 in the 760→760 treatment. Since these were batch cultures, the pH of the media increased steadily and after 4 d the 760 µatm pCO_2_ culture had changed from an initial pH of 7.9 to 8.7±0.1 and the air equilibrated cultures had increased from pH 8.1 to 9.1±0.1. Although there were slight and consistent differences between cell densities in the different treatments, they were not statistically significant. PERMANOVA showed no differences between treatments or between specific growth rates at 3 points on the growth curve or on any interaction between time point and treatment.

In contrast to the long-term continuous cultures, there were no significant differences in a number of physiological measures. The C:N ratios of the batch cultures under the 4 treatments (760→760, 760→380, 380→760 & 380→380 µatm) were not significantly different (global test ANOSIM: R = 0.03, P = 19.5%). The mean (±SD) C:N ratios were 6.14±0.15 for 760→760, 6.31±0.52 for 760→380, 5.95±0.25 for 380→760 and 6.17±0.23 for 380→380 cultures. In addition, photosynthetic parameters were uniform in these experiments. A PERMANOVA analysis detected no significant differences between the 4 treatments in either F_v_/F_m_ or σ. Mean F_v_/F_m_ ratios were 0.59±0.02 (760→760), 0.58±0.03 (760→380), 0.60±0.02 (380→760) and 0.59±0.01 (380→380). Values of σ were 856±40 (760→760), 908±98 (760→380), 829±37 (380→760), and 877±19 (380→380). Finally, there were no significant differences in forward scatter, side scatter or chlorophyll fluorescence cell^−1^ in cultures grown under different CO_2_ treatments (Table 2).

**Table 2:** Test for acclimation or adaptation of *T. pseudonana* after 3 months continuous culture in media at 760 µatm pCO_2_ or present-day conditions of 380 µatm pCO_2_.

	760→760	760→380	380→760	380→380
**Red fl cell^−1^**	360±6	317±16	356±41	350±52
**SSC cell^−1^**	60±6	55±4	56±8	59±13
**FSC cell^−1^**	52±3	47±3	51±6	51±8
**Fv/Fm**	0.59±0.02	0.58±0.03	0.60±0.02	0.59±0.01
**σ**	856±40	908±98	829±37	877±19
**C:N ratio**	6.14±0.15	6.31±0.52	5.95±0.25	6.17±0.23

760→760 - cells from the long-term culture grown at 760 µatm were used to inoculate a batch culture equilibrated with 760 µatm CO_2_ aq media: 760→380 - the same cells (760) inoculated culture media equilibrated with air (380): 380→760 - cells from long-term culture equilibrated with air were inoculum for a batch culture equilibrated with 760 µatm CO_2_ aq media: 380→380 – air grown cells inoculate air-equilibrated batch culture. All data are means (±SD) evaluation experiment. Flow cytometry readings for red fluorescence (Red fl), side scatter (SSC) and forward scatter (FSC) per cell, determinations of Fv/Fm, σ, C:N ratio.

## Discussion

It is essential to understand how phytoplankton will respond to the higher CO_2_ (aq)/lower pH conditions that will occur in the surface ocean in the near future as a result of the dissolution of anthropogenic CO_2._ However, this study highlights the difficulty of investigating the effects of ocean acidification in laboratory culture. Cell densities that are commonly employed in culture rapidly deplete CO_2_ (aq) from the medium and result in rapid increases in pH. For example, Shi and colleagues [19] observed that *T. weissflogii* cells growing to a cell density of 5×10^3^ cells mL^−1^ from an initial inoculum of 20–100 cells mL^−1^ caused an increase of 0.1 pH units. Buffers can help to maintain target pH, but there are often unknown effects that make them less than ideal and, in this study, resulted in lower specific growth rates.

As we demonstrate here, dilute cell densities provide one approach that allows long-term maintenance of target pH. The approach adopted here, of dilution with media equilibrated with different pCO_2_, has advantages over other designs of pH stat that are maintained by the addition of acid or base in response to deviations from the required pH. Although addition of acid or base is very effective at maintaining pH, total alkalinity may be altered, which at high biomass can lead to significant perturbations in carbonate chemistry. However, a disadvantage of the dilution method of the pH auxostat is that it is highly labour-intensive, and large volumes of pre-equilibrated media are required to maintain the cultures.

Overall, there were very few effects on *T. pseudonana* of long-term culture at different pCO_2_ and pH (Table 1). Certainly, neither forward nor side scatter measurements were able to detect any difference between cells grown at higher or present-day CO_2_ (aq). However, there were significant differences in C:N ratio and in red fluorescence cell^−1^; C:N ratio was significantly lower and red fluorescence cell^−1^ was elevated in the higher CO_2_ (aq) cultures (although there was high variability between replicate cultures). In fact, the C:N ratio of the higher CO_2_ (aq) culture was closer to the Redfield ratio of 6.6 than for the cells from the present-day CO_2_ (aq) cultures. Changes in C:N ratio and increased production of extracellular organic matter have been reported in other studies of the effect of ocean acidification and the term ‘carbon overconsumption’ has been applied to the uptake of carbon in excess of the expected amount calculated from the nitrate uptake and Redfield ratio [35]–[38]. In the present study, extracellular production was not measured and, although there were significant differences in C:N ratio between treatments, this was largely the result of high variability in one of the replicate cultures. In this study, we have no evidence for consistent increases in C content after >100 generations growth at higher CO_2_ (aq).

Red fluorescence cell^−1^ was significantly higher after >100 generations at 760 µatm than at 380 µatm pCO_2_ (F = 5.23, P<0.05: Table 1). Other studies have also suggested that increased CO_2_ can lead to higher pigment content per cell in phytoplankton [22], [38]. However, variation in pigment content is usually associated with photo-acclimation, rather than changes in DIC [21]. Whatever the reason, higher red fluorescence cell^−1^ did not result in detectible changes to photophysiology. Determinations of Fv/Fm and σ_PSII_ were not significantly different in any of the treatments. There is no evidence from our measurements to suggest that photosynthetic rate had changed during the long-term culture period.

We also have no evidence that growth rate varied as a consequence of CO_2_ treatment. In semicontinuous cultures, growth rates were identical in cultures supplied with 780 or 360 µatm CO_2_ (aq). Other studies have also failed to find a direct effect of elevated CO_2_ on cell division of *T. pseudonana*. Pruder and Bolton [10] found that growth rate was unaffected by increased CO_2_ at 760 µatm pCO_2_, and similar results have been reported for other diatom species [13], [15], [19], [39].

The most significant effect of culture treatment that we have measured was in expression of CA. There are 13 candidate sequences in the *T. pseudonana* genome, which are predicted to encode 3 α-, 5 γ-, 4 δ- and 1 ξ-CAs although to date none have been characterized experimentally for CA enzyme activity [31]. McGinn and Morel [26] demonstrated that, of the four δ-CAs in *T. pseudonana*, *CA-4* and *CA-6* transcripts were the most abundant and the most responsive to changes in carbon availability; it should be noted that very high (1% - 10000 µatm) pCO_2_ (aq) concentrations were used to demonstrate the regulation of δ-CA expression [26]. *CA-4* and *CA-6* were strongly induced by CO_2_ depletion (pH 8.9) and highly repressed in the presence of 1% CO_2_ at pH 7.2 [26]. Tachibana and colleagues [31] examined the expression of three CAs by RT-PCR and found that transcripts of *CA-1*, *CA-3* and *CA-7* (representing α-, ξ- and δ-CAs respectively) were much less abundant in cells grown at 0.16% (1600 µatm pCO_2_) compared to cells grown in media equilibrated with air. In the present study, which used a much lower *p*CO_2_ – 780 or 360 µatm CO_2_ (aq) in long-term cultures – the effect of treatment on δ-CA transcription was more subtle. Only *CA-4* showed any significant change and was substantially repressed at higher CO_2_. We do not know the subcellular localisation of the δ-CAs in *T. pseudonana*. Using signal sequence analysis, Tachibana and colleagues [31] predicted that the δ-CAs would be located in the cytosol, but did not test this experimentally. However, by expressing GFP-fusions, they were able to demonstrate that *CA-1* and *CA-3* were localized to the stroma and the cytosol respectively. As both of these CAs exhibited reduced transcription in cells grown at high CO_2_, we might expect that multiple aspects of the CCM in *T. pseudonana* will respond to future elevations in atmospheric CO_2_.

The transcriptional responses of *CA-4* were measured after ∼100 generations of growth at 780 µatm CO_2_ (aq); so this was not a transient response to changing pCO_2_, but persisted throughout a long-term acclimation. Only one component of CCMs was investigated in this study and no doubt there would be many differences in the transcriptome of *T. pseudonana* between growth at 780 or 360 µatm CO_2_ (aq). The only other gene investigated in this study, RUBISCO small subunit, showed no variation in *rbcS* transcription between CO_2_ treatments. This is consistent with the findings of Granum and colleagues [40] that RUBISCO transcription is rather invariant, since there were no differences in expression of the large RUBISCO subunit (*rbcL*) in *T. pseudonana* cultures grown at 100 or 380 µatm CO_2_.

So, if higher pCO_2_ were to be beneficial to phytoplankton in a future high-CO_2_ ocean, where would significant savings in protein costs occur? That is, would there be significant benefits to the cell by modulating CO_2_ supply to RUBISCO rather than the regulating the expression levels of the enzyme? RUBISCO comprises a large fraction of total protein in cyanobacteria and microalgae (from 4% to 0.23% of total cell protein [6], [41]). CAs are probably a much smaller proportion of total protein – periplasmic CAs are of the order of 0.0012% in *Chlamydomonas reinhardtii* [42]. A reduction in synthesis of the CO_2_-responsive CAs in *T. pseudonana* is unlikely to have much impact on the protein costs of the cell. However, a recent study of the efficiency of the CCM in marine diatoms, which integrates a physiological and a modelling approach, suggests that active transport of HCO_3_^-^ into the chloroplast constitutes the major energetic cost of the CCM [43]. A model of the *Phaeodactylum tricornutum* CCM, which ignores the comparatively minor costs relating to the synthesis of CCM components, predicts that a doubling of atmospheric CO_2_ could result in a 20% reduction in the energetic cost of the CCM, equating to a 3–6% reduction in the total cost of carbon fixation [43]. Thus, the doubling of atmospheric CO_2_ from present-day levels may yield an energetic benefit to diatoms, but any benefits would be marginal and may not be detectable as a change in specific growth rate. This prediction is in keeping with the results of our long-term experimental study of *T. pseudonana* at elevated CO_2_, in which we observed little change in multiple physiological parameters. As this study has shown, standard methods to determine µ_max_ do not have the sensitivity to detect any subtle variations in response to different pCO_2_ concentrations. The labour-intensive nature of the pH auxostat cultures meant that replication was limited to 3 cultures per treatment and we are aware that this limits the power of our statistical analyses. Performing long-term experiments under carefully controlled conditions, with sufficient replication to allow statistical determination of subtle phenotypes, has been recognised as an important challenge [23].

In conclusion, growth over 100 generations did not provide evidence for significant adaptation of *T. pseudonana* CCMP1335 to increased CO_2_. There were small changes in C:N ratio and reduced expression of one δ-CA when cells were maintained at increased CO_2_ for 3 months. *T. pseudonana* appears to be capable of successful acclimation to growth at a wide range of pH. Although data from other diatom species are currently lacking, if all diatoms respond in a similar fashion to *T. pseudonana*, acidification of this magnitude in the future ocean may have little effect on diatom productivity.

## Supporting Information

Figure S1 **Design of a single vessel for pH auxostat continuous culture.** Cultures were bubbled continuously with air at either 760 µatm pCO_2_ or 380 µatm pCO_2_. Cultures were also stirred and pH was continuously monitored via a pH meter. An increase in pH triggered the pump, resulting in an influx of f/2 seawater media which was pre-equilibrated via aeration with air at either 760 µatm pCO_2_ or 380 µatm pCO_2._ The media influx dilutes the culture, acting to both reduce the cell density and to restore the original pH. Six of these culture vessels were maintained attached to a single control unit.
(TIF)

Table S1 **Primer sequences for qRT-PCR.** Forward (F) and reverse (R) primer pairs were designed to amplify regions of 200–300 bases for real-time PCR analysis. The alternative gene identity refers the name used by McGinn & Morel [23]. CA-7 has been previously identified by the protein ID 22619, although protein ID 34094 is used in the genome.
(DOC)
