# Supplementary figures and images for: The Response of Thalassiosira pseudonana to Long-Term Exposure to Increased CO2 and Decreased pH
Source: PLoS One. 2011 Oct 28;6(10):e26695. doi: 10.1371/journal.pone.0026695 (PMC3203894; doi:10.1371/journal.pone.0026695)

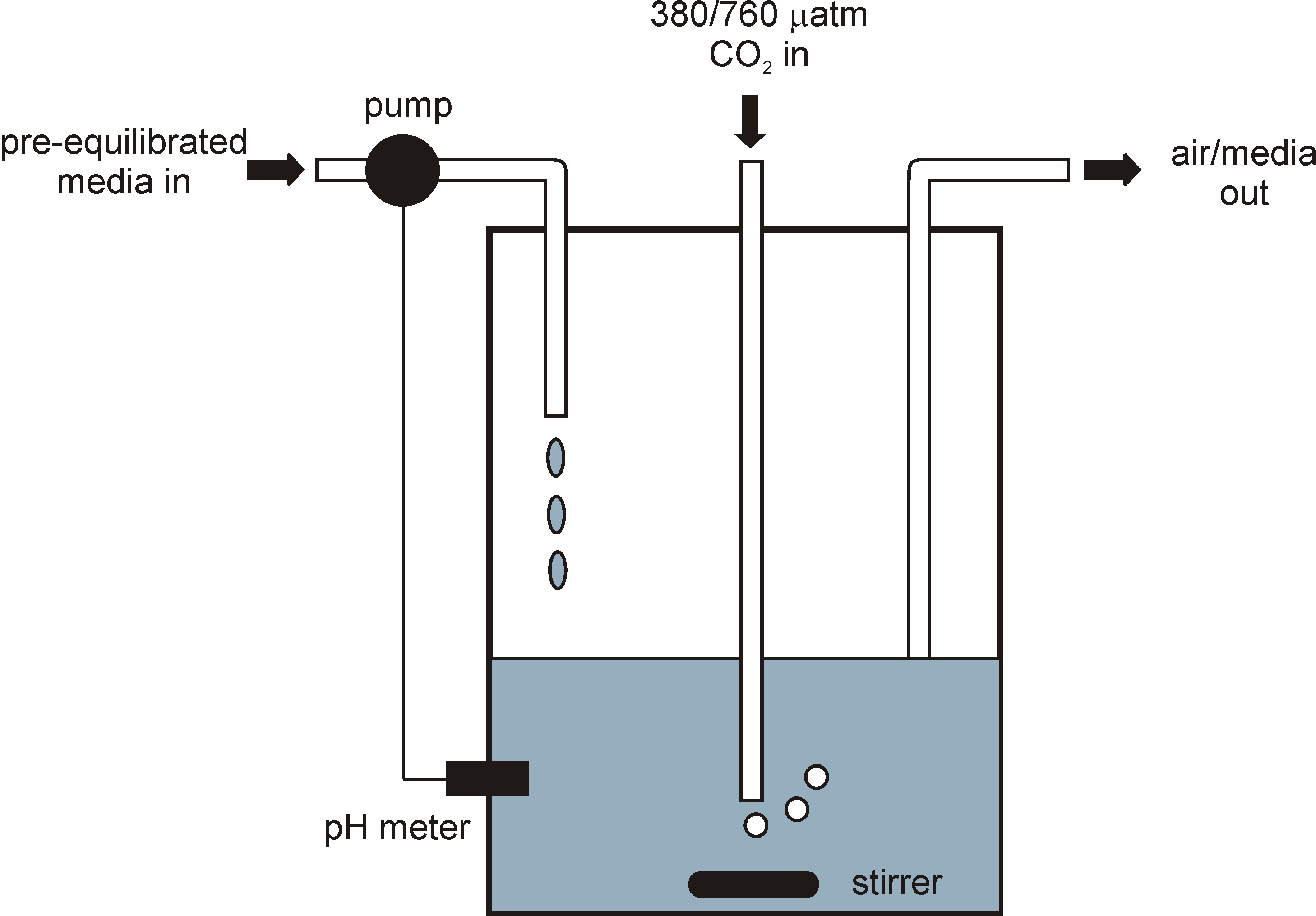

Supplement: Figure S1 — Design of a single vessel for pH auxostat continuous culture. Cultures were bubbled continuously with air at either 760 µatm pCO2 or 380 µatm pCO2. Cultures were also stirred and pH was continuously monitored via a pH meter. An increase in pH triggered the pump, resulting in an influx of f/2 seawater media which was pre-equilibrated via aeration with air at either 760 µatm pCO2 or 380 µatm pCO2. The media influx dilutes the culture, acting to both reduce the cell density and to restore the original pH. Six of these culture vessels were maintained attached to a single control unit. (TIF) [file pone.0026695.s001.tif]
