# Supplementary material for: The Response of Thalassiosira pseudonana to Long-Term Exposure to Increased CO2 and Decreased pH
Source: PLoS One. 2011 Oct 28;6(10):e26695. doi: 10.1371/journal.pone.0026695 (PMC3203894; doi:10.1371/journal.pone.0026695)
Supplement: Table S1 — Primer sequences for qRT-PCR. Forward (F) and reverse (R) primer pairs were designed to amplify regions of 200–300 bases for real-time PCR analysis. The alternative gene identity refers the name used by McGinn & Morel [23]. CA-7 has been previously identified by the protein ID 22619, although protein ID 34094 is used in the genome. (DOC) [file pone.0026695.s002.doc]

**Table S1**

| **Gene** | **Alternative name** | **Protein ID** |  | **Sequence 5’-3’** | **T_m_ (^o^C)** | **GC (%)** |
| --- | --- | --- | --- | --- | --- | --- |
| ***EF1α*** |  |  | F | -GTA TCG GCA CTG TCC CTG TT- | 59.4 | 55 |
|  |  |  | R | -ATC TCC TCG CCG AAT ATC CT- | 59.4 | 55 |
| **Actin** |  |  | F | -ACG TGA CCT CAC GGA CTA CC- | 61.4 | 60 |
|  |  |  | R | -CAG TAA GGT TGG GCT GGA AA- | 57.3 | 50 |
| ***rbcS*** |  |  | F | -TAA AGG CTG GGC TAT GAA CG- | 57.3 | 50 |
|  |  |  | R | -AAC CTG GCT CAT TAG CTG GA- | 57.3 | 50 |
| ***CA-4*** | *TWCAtp1* | 233 | F | -CAG TGA TGG TGC AGC TGT TG- | 59.4 | 55 |
|  |  |  | R | -CGC CAA GCG TAG TGA AAC TC- | 59.4 | 55 |
| ***CA-5*** | *TWCAtp4* | 814 | F | -GAA CAG CGT CAA CAA CAA CC- | 57.3 | 55 |
|  |  |  | R | -AAC TTC TCC TCA TCC AAG TTG C- | 57.3 | 50 |
| ***CA-6*** | *TWCAtp2* | 34125 | F | -ACG GTG ACG GTC CCC ACG GTA ACATC- | 69.5 | 61.5 |
|  |  |  | R | -ACC CAC AGC AGA GGC GAT ATC CTG A- | 66.3 | 56 |
| ***CA-7*** | *TWCAtp3* | 34094 | F | -ATG CGT CTG CAG AAG AAG AG- | 59.4 | 55 |
|  |  | (22619) | R | -TGC TCT CCC AAT GCA TTA AA- | 53.2 | 40 |
